# Nationwide molecular surveillance of three *Plasmodium* species harboured by symptomatic malaria patients living in Ghana

**DOI:** 10.1186/s13071-022-05153-6

**Published:** 2022-01-28

**Authors:** Linda E. Amoah, Kwame K. Asare, Donu Dickson, Sherik-fa Anang, Abena Busayo, Dorcas Bredu, George Asumah, Nana Peprah, Alexander Asamoah, Benjamin Abuaku, Keziah L. Malm

**Affiliations:** 1 Department of Immunology, Noguchi Memorial Institute for Medical Research, University of Ghana, Accra, Ghana; 2 Department of Biomedical Science, School of Allied Health Sciences, College of Allied Health Sciences, University of Cape Coast, Cape Coast, Ghana; 3 National Malaria Control Program, Accra, Ghana; 4 Department of Epidemiology, Noguchi Memorial Institute for Medical Research, University of Ghana, Accra, Ghana; 5 Department of Nutrition, Noguchi Memorial Institute for Medical Research, University of Ghana, Accra, Ghana

**Keywords:** Symptomatic Malaria, *Plasmodium falciparum*, *Plasmodium malariae*, *Plasmodium ovale*, Malaria prevalence, Ghana

## Abstract

**Background:**

Clinical presentations of malaria in Ghana are primarily caused by infections containing microscopic densities of *Plasmodium falciparum*, with a minor contribution from *Plasmodium malariae* and *Plasmodium ovale*. However, infections containing submicroscopic parasite densities can result in clinical disease. In this study, we used PCR to determine the prevalence of three human malaria parasite species harboured by suspected malaria patients attending healthcare facilities across the country.

**Methods:**

Archived dried blood spots on filter paper that had been prepared from whole blood collected from 5260 patients with suspected malaria attending healthcare facilities across the country in 2018 were used as experimental material. *Plasmodium* species-specific PCR was performed on DNA extracted from the dried blood spots. Demographic data and microscopy data for the subset of samples tested were available from the original study on these specimens.

**Results:**

The overall frequency of *P. falciparum*, *P. malariae* and *P. ovale* detected by PCR was 74.9, 1.4 and 0.9%, respectively. Of the suspected symptomatic *P. falciparum* malaria cases, 33.5% contained submicroscopic densities of parasites. For all regions, molecular diagnosis of *P. falciparum*, *P. malariae* and *P. ovale* was significantly higher than diagnosis using microscopy: up to 98.7% (75/76) of *P. malariae* and 97.8% (45/46) of *P. ovale* infections detected by PCR were missed by microscopy.

**Conclusion:**

*Plasmodium malariae* and *P. ovale* contributed to clinical malaria infections, with children aged between 5 and 15 years harbouring a higher frequency of *P. falciparum* and *P. ovale*, whilst *P. malariae* was more predominant in individuals aged between 10 and 20 years. More sensitive point-of-care tools are needed to detect the presence of low-density (submicroscopic) *Plasmodium* infections, which may be responsible for symptomatic infections.

**Graphical Abstract:**

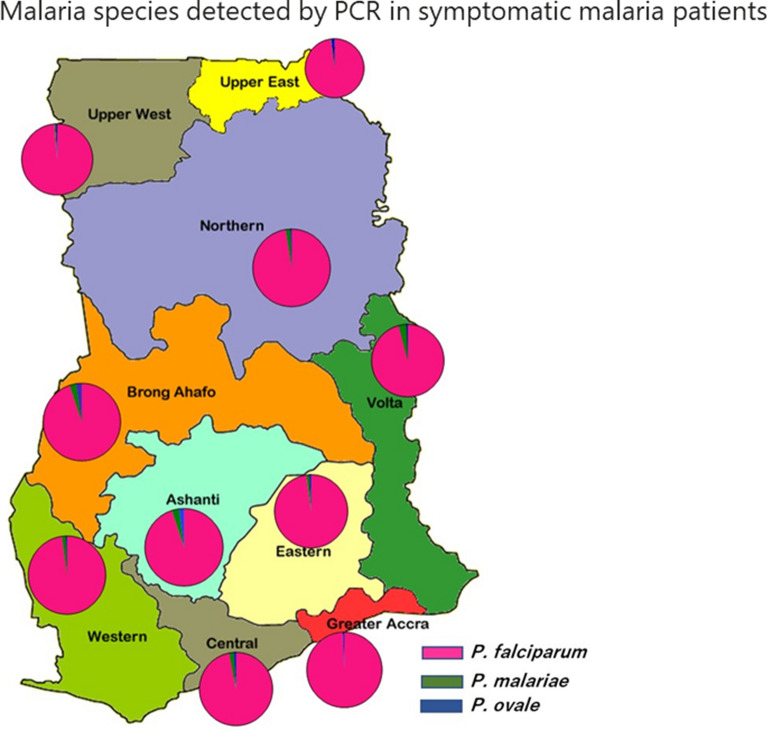

**Supplementary Information:**

The online version contains supplementary material available at 10.1186/s13071-022-05153-6.

## Background

*Plasmodium* species that infect humans include *Plasmodium malariae*, *Plasmodium ovale*, *Plasmodium vivax*, *Plasmodium knowlesi*, and *Plasmodium falciparum*; of these  *P. falciparum* is responsible for most of the malaria cases in Ghana [1, 2]. In Ghana, low-level infections caused by *P. malariae* and *P. ovale* have been reported, but these species are predominantly present as mixed infections with *P. falciparum* [3, 4] and also as low-density infections.

Infections with all human *Plasmodium* parasites can result in various presentations of disease, with the uncomplicated and asymptomatic presentations being the most predominant manifestations of parasite infection. Uncomplicated malaria often presents with fever, chills, profuse sweating, headache, vomiting and diarrhoea [2], whilst asymptomatic infections are devoid of apparent physical symptoms of the disease. Some infections, however, do result in severe disease, whose presentations include severe anaemia and end-organ damage such as cerebral malaria, pulmonary complications and hypoglycaemia [5].

Microscopy is the gold standard for *Plasmodium* parasite detection, especially for clinical cases of malaria as these usually contain high densities of parasites [6, 7]. Microscopy can provide an estimate of malaria parasite density from thick blood smears and information on the various parasite species present in an infection from a thin blood smear. The downside to microscopy is that it requires experts to prepare and stain the blood smear as well as electricity and a microscope, in addition to an expert malaria microscopist to read and grade the smear.

Accurate estimation of the density of the infecting parasites and accurate identification of the infecting parasite species are two properties of infection that are essential to the proper management of malaria, including providing information on the appropriate drug treatment [8, 9]. In malaria endemic areas where* P. falciparum* is predominant and widespread, *P. malariae*, and *P. ovale* infections are usually underdiagnosed, with almost all clinical malaria cases attributed to *P. falciparum* infection [10, 11]. Some factors that have been associated with underdiagnosing non-*falciparum* malaria by microscopy in *P. falciparum* malaria endemic countries include: (i) the very low parasitaemia of *P. malariae* and *P. ovale*; and (ii) the lack of highly skilled microscopists who can distinguish the different *Plasmodium* species [12–14]. Molecular tools, including various forms of nucleic acid amplification tests, such as conventional and real-time PCR, can provide accurate estimates of parasite density and accurate identification of the infecting parasite species. PCR methodology is not a point-of-care diagnostic tool and is typically reserved for research in malaria-endemic countries; however, it is regarded as the new gold standard in cases of low parasitemia and mixed-species infections [5]. The sensitivity and selectivity of PCR methods are superior to those of microscopy and rapid diagnostic tests, with the detection limit of nested PCR set at as low as 0.2 parasite/µl [15].

Ghana is a malaria mesoendemic country which still has some facilities to confirm the suspicion of malaria in patients suspected of having this disease (symptomatic individuals) with microscopy. The aim of this study was to use PCR to determine the frequency and distribution of the three common malaria parasite species in patients with suspected malaria who were seeking treatment in 100 different healthcare facilities across Ghana in 2018.

## Methods

### Study population

Convenience sampling was used to select between 292 and 600 archived dried blood spots on filter paper from a pool of about 2000 such samples that had been collected in the original study from patients with suspected malaria attending clinics in the 10 regions of Ghana. The adoption of convenience sampling limited the data for analysis to the selected samples, with the inherent possibility of under-/over-representation of the original study population. The selection criterion for the samples was any dried blood spot on filter paper for which there was sufficient material to punch out two 3-mm-radius spots for analysis. The samples used originated from samples taken from 5260 individuals who had been recruited as part of a larger study that enrolled 19,787 patients with suspected symptomatic malaria. Patients for this larger study were recruited from 100 healthcare facilities (10 from each of the then 10 regions of the country). Study participants ranged in age from 1 to 94 years. The demographics of the larger study population have been reported previously [16, 17].

### Study site

This cross-sectional study was conducted in the 10 regions of Ghana as of 2018: Greater Accra Region (GAR), Central Region (CR), Ashanti Region (AR), Western Region (WR), Eastern Region (ER), Brong-Ahafo Region (BAR), Volta Region (VR), Northern Region (NR), Upper East Region (UER) and Upper West Region (UWR). Ten healthare facilities were randomly selected in each of the 10 regions of Ghana [16, 17].

### Sample collection and processing

The samples used in this study were collected between May and August 2018 as described earlier [16, 17]. Demographic data were obtained from each consenting participant, and about 2 ml of venous blood was collected into an EDTA-vacutainer® tube. Thick and thin blood smears were prepared and processed for malaria parasite identification and quantification as previously described [2]. Dried blood spots on filter paper were prepared by spotting four 50-µl drops of the venous blood onto a Whatman® No. 3 filter paper (Whatman plc, Maidstone, UK), following which the blood spots were air-dried and stored individually in zip-lock bags containing silica gels. Packed cells were then isolated from the remaining venous blood and stored in a 1.5-ml Eppendorf tube at − 20 °C for future use [16].

### Microscopy

Each thick and thin blood smear obtained from the original study was read by two independent WHO-certified malaria microscopists; any disagreement on smear readings was resolved by re-examination and assessment by a third microscopist. The assessment by the third microscopists was considered to be the final decision [16, 17].

### DNA extraction

Genomic DNA was extracted from the dry blood spots (DBS) using the Chelex extraction method [18, 19] with few modifications. Each punched-out DBS was placed into a sterile 1.5-ml microfuge tube containing 1 ml of 1× phosphate-buffered saline (PBS) supplemented with Tween-20 solution (0.5% PBST). The tubes were incubated overnight at room temperature with intermittent shaking, then centrifuged for 2 min at 21,000 *g* and the reddish supernatant decanted. The DBS punches were then washed in ice-cold PBS at 4 °C for 30 min and the supernatant again discarded. Finally, 50 μl of freshly prepared 20% Chelex-100 in distilled water and 100 μl of distilled water were added to each tube. The tubes were then heated at 95 °C for 10 min, with repeated vortexing at 2-min intervals. The tubes containing the extracted DNA were finally centrifuged at 21,000 *g* for 8 min, and the supernatant containing the DNA was transferred into a new sterile tube and stored at – 20 °C until use.

### Nested PCR

#### *Plasmodium* species (*P. falciparum*, *P. malariae*, and *P. ovale*) identification

The *18S* ribosomal RNA gene, a conserved region in *P. falciparum*, *P. malariae* and *P. ovale*, was amplified using nested PCR from genomic DNA using a protocol described previously [2], with minor modifications. In the primary reaction (nest 1), 250 nM of the genus-specific primers rPLU1 (forward) and rPLU5 (reverse) were used in a total reaction volume of 10 μl consisting of of 2 μl DNA template, 1× PCR buffer, 200 nM dNTPs, 700 nM MgCl2 and 1 U of OneTaq DNA polymerase. In the secondary reaction (nest 2), the species-specific primers rFAL1/rFAL2 (200 nM), rMAL1/ rMAL2 (200 nM) and rOVA1/rOVA2 (200 nM) were used in separate 10-μl reactions using 0.5 μl of the primary reaction product as a template for the identification of *P. falciparum*, *P. malariae* and *P. ovale*, respectively. The primary and secondary reactions were run at an initial denaturation temperature of 94 °C for 2 min; this was followed by a second denaturation at 94 °C for 30 s, primer annealing at 55 °C (for initial reaction) and 58 °C (for the second reaction), both for 30 s, and extension at 68 °C for 1 min 30 s for nest 1, 30 s for nest 2 (*P. falciparum*, *P. malariae*) and 1 min for *P. ovale* (nest 2); and a final extension at 68 °C for 5 min. Positive control samples used for this study included known *P. falciparum* and *P. ovale* or *P. malariae* DNA samples that had been obtained as part of the Distribution 4881 of the WHO Malaria NAAT EQA programme; ultra-pure water was used as a negative control. The nest 2 PCR products were resolved in a 2% agarose gel pre-stained with ethidium bromide. The gels were subsequently visualised using the UV settings on the Vilber gel documentation system (Vilber, Collégien, France).

### Statistical analysis

All data acquired during this study were entered into Microsoft Excel (Microsoft Corp., Redmond, WA, USA), and the statistical analyses were performed with GraphPad Prism software, version 8.4.3 (GraphPad Software, San Diego, CA, USA). The data were grouped according to regions, diagnostic tests, gender and age categories. The frequency of malaria species was determined using simple counts and proportions. The associations between frequency of species and explanatory variables were determined using chi-square statistics. Statistical significance was set at *P* < 0.05.

## Results

### Demographic characteristics of study subjects

The distribution of three different *Plasmodium* species in the 10 regions of the country was assessed in all dried blood spot punches from 5260 study subjects with suspected malaria. The mean age ± standard error of the mean of these subjects in the study year (2018) ranged from 14.2 ± 0.7 (95% confidence interval [CI]: 12.8–15.5) in the Brong Ahafo region to 27.2 ± 0.9 (95% CI: 25.5–29.0) in the Upper West region of Ghana. Female participants slightly outnumbered male ones across all regions; the Central region had the highest proportion of female participants (62.9%) (Table 1).

**Table 1:** Demographic characteristics of the study subjects according to region of Ghana

Demographic characteristics	Region of Ghana^a^										
	AR	BAR	CR	ER	GAR	NR	UWR	UER	VR	WR	Total
Age, years, mean ± SEM (95% CI)	16.91 ± 0.74 (15.44–18.37)	14.15 ± 0.70 (12.78–15.52)	20.03 ± 0.76 (18.54–21.51)	23.32 ± 1.21 (20.96–25.76)	24.73 ± 0.73 (23.32–26.13)	18.81 ± 0.73 (17.38–20.24)	27.22 ± 0.89 (25.46–28.97)	19.65 ± 0.96 (17.75–21.55)	18.99 ± 0.89 (17.24–20.73)	16.67 ± 0.72 (15.25–18.08)	20.10 ± 0.26 (19.58–20.61)
Sex (female/male),* n*	508 (286/222)^c^	560 (319/241)^c^	607 (382/225)	288 (166/122)^c^	617 (346/271)^c^	597 (347/250)^c^	594 (391/203)^c^	400 (229/171)^c^	507 (295/212)	443 (260/183)^c^	5121 (3021/2100)
PCR results,* n*/*N* (%)											
*Plasmodium falciparum*-positive	497/519 (95.76)	523/563 (92.89)	493/607 (81.21)	235/292 (80.48)	344/625 (55.04)	407/600 (67.83)	304/655 (46.41)	331/408 (81.13)	430/507 (84.81)	378/484 (78.10)	3942/5260 (74.94)
*Plasmodium malariae*-positive	15/519 (2.89)	15/563 (2.66)	11/607 (1.81)	3/292 (1.03)	0/625 (0.00)	8/600 (1.33)	2/655 (0.31)	1/408 (0.25)	15/507 (2.96)	6/484 (1.24)	76/5260 (1.44)
*Plasmodium ovale*-positive	11/519 (2.12)	13/563 (2.31)	3 /607 (0.50)	2/292 (0.68)	2/625 (0.32)	2/600 (0.33)	2/655 (0.31)	6/408 (1.47)	3/507 (0.59)	2/484 (0.41)	46/5260 (0.87)
Microscopy,* n*/*N* (%)											
Microscopy-positive	412/476 (86.55)^d^	335/563 (59.50)	295/512 (57.62)^d^	41/292 (14.04)	132/570 (23.16)^d^	173/543 (31.86)^d^	155/618 (25.08)^d^	189/408 (46.32)	277/492 (56.30)^d^	277/463 (59.83)^d^	2286/4937 (46.30)
Microscopy-negative-	64/476 (13.45)	228/563 (40.50)	217/512 (42.38)	251/292 (85.96)	438/570 (76.84)	370/543 (68.14)	463/618 (74.92)	219/408 (53.68)	215/492 (43.70)	186/463 (40.17)	2651/4937 (53.70)
Submicroscopy^b^,* n*/*N* (%)											
*P. falciparum*	85/497 (17.10)	188/523 (35.95)	198/493 (40.16)	194/235 (82.55)	212/344 (61.63)	234/407 (57.49)	149/304 (24.11)	142/331 (42.90)	153/430 (35.58)	101/378 (26.71)	1656/3942 (42.01)
*P. malariae*	14/15 (93.33)	15/15 (100.00)	11/11 (100.00)	3/3 (100.00)	0 (0.00)	8/8 (100.00)	2/2 (100.00)	1/1 (100.00)	15/15 (100.00)	6/6 (100.00)	75/76 (98.68)
*P. ovale*	10/11 (90.91)	13/13 (100.00)	3/3 (100.00)	2/2 (100.00)	2/2 (100.00)	2/2 (100.00)	2/2 (100.00)	6/6 (100.00)	3/3 (100.00)	2/2 (100.00)	45/46 (97.83)

*CI *Confidence interval, * SEM* standard error of the mean; *n* positive samples, *N* total samples tested

^a^AR, Ashanti region; BAR, Brong Ahafo region; CR, Central region; ER, Eastern region; GAR, Greater Accra region; NR, Northern region; UER, Upper East region; UWR, Upper West region; VR, Volta region; WR, Western region

^b^Submicroscopy is defined as PCR-positive but microscopy-negative: formula = ([PCR-positive samples − microscopy-positive samples])/PCR-positive samples) × 100

^c^Missing gender data among the selected samples

^d^Missing microscopic data among the selected samples used for PCR diagnosis

### Regional distribution of *Plasmodium* species among study subjects

#### Microscopy

The frequency of *P. falciparum* detected by microscopy in the subset of samples used in this study was 2284/4937 (46.3%), with the highest frequency identified in the Ashanti region 412/476 (86.6%) and the lowest detected in the Eastern region 41/292 (14.0%) (Table 1). Microscopy identified one *P. malariae* and one *P. ovale* infection, which were both observed in the Ashanti region. A few samples (323/5260, 6.1%) were missing microscopy data.

#### PCR

**Fig. 1:**
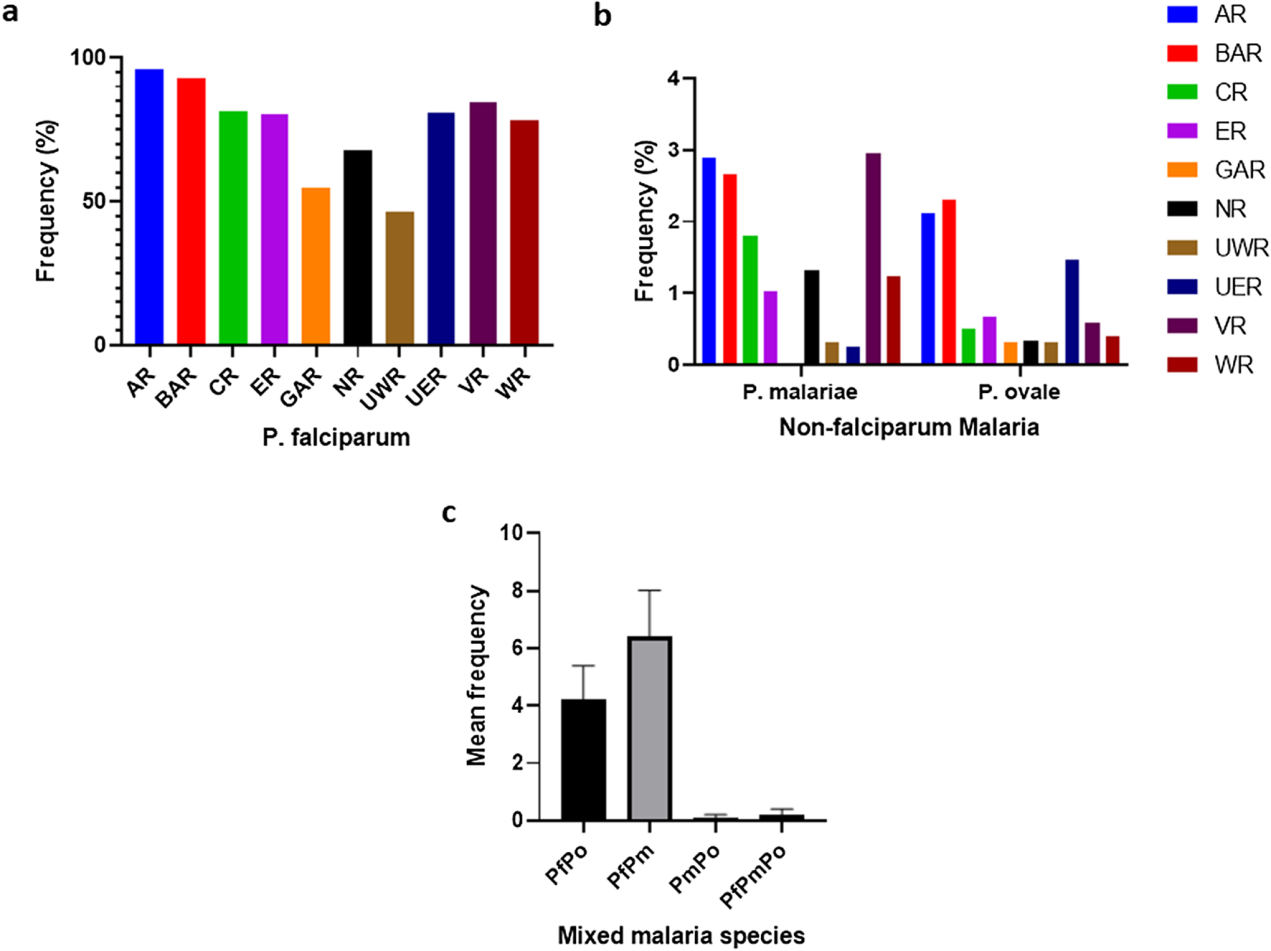
The prevalence of *Plasmodium falciparum* and non-*falciparum* parasites in 10 regions of Ghana. **a** Regional prevalence of *P. falciparum* detected by PCR in Ghana. **b** Prevalence of non-*falciparum* malaria (*Plasmodium malariae* and *Plasmodium ovale*) detected by PCR across the 10 regions of Ghana. **c** Bar graph showing the prevalence of mixed *Plasmodium* infections in Ghana. Abbreviations (**a**, **b**): AR, Ashanti region; BAR, Brong Ahafo region; CR, Central region; ER, Eastern region; GAR, Greater Accra region; NR, Northern region; UER, Upper East region; UWR, Upper West region; VR, Volta region; WR, Western region. Abbreviations (**c**): Pf, *Plasmodium falciparum*; Pm, *Plasmodium malariae*; Po, *Plasmodium ovale*

Overall, PCR detected *P. falciparum* in 3942 of the 5260 (74.9%) individuals sampled, with the highest frequency identified in the Ashanti region (497/519, 95.8%) and lowest detected in the Upper West region (304/655, 46.4%) (Fig. 1a; Table 1). There was a significant difference in the frequency of *P. falciparum* detected by PCR compared to microscopy in all the regions of Ghana (*χ*^2^ = 215.24, *df* = 1, *P* < 0.0001) (Table 2).

**Table 2:** Chi-square statistics comparing the prevalence of *Plasmodium* species by PCR and microscopy at each of the 10 regions of Ghana

Regions	Diagnostics	*P. falciparum*			*df*	*P*	*P. malariae*			*df*	*P*	*P. ovale*			*df*	*P*
		Detection frequency					Detection frequency					Detection frequency				
		Positive (+)	Negative (−)	*χ*^2^			Positive ( +)	Negative (−)	*χ*^2^			Positive ( +)	Negatve (−)	*χ*^2^		
AR	PCR	497	22	28.54	1	< 0.0001	15	504	11.27	1	0.0008	11	508	7.6	1	0.0058
	Microscopy	410	66				1	475				1	475			
BAR	PCR	523	40	173.1	1	< 0.0001	15	548	15.2	1	< 0.0001	13	550	13.15	1	0.0003
	Microscopy	335	228				0	563				0	563			
CR	PCR	493	114	74.27	1	< 0.0001	11	596	9.37	1	0.0022	3	604	2.54	1	0.1112
	Microscopy	295	217				0	512				0	512			
ER	PCR	235	57	258.6	1	< 0.0001	3	199	4.36	1	0.0367	2	290	2.01	1	0.1566
	Microscopy	41	251				0	292				0	292			
GAR	PCR	344	281	126.4	1	< 0.0001	0	625	-		-	2	623	1.83	1	0.1765
	Microscopy	132	438				0	570				0	570			
NR	PCR	407	193	147.6	1	< 0.0001	8	592	7.29	1	0.0069	2	598	1.81	1	0.1781
	Microscopy	173	370				0	543				0	543			
UWR	PCR	304	351	62.76	1	< 0.0001	2	653	1.89	1	0.1692	2	653	1.89	1	0.1692
	Microscopy	155	463				0	618				0	618			
UER	PCR	331	72	113	1	< 0.0001	1	407	1	1	0.317	6	402	6.04	1	0.014
	Microscopy	189	219				0	408				0	408			
VR	PCR	430	77	98.13	1	< 0.0001	15	492	14.78	1	< 0.0001	3	504	2.92	1	0.0875
	Microscopy	277	215				0	492				0	492			
WR	PCR	378	106	37.04	1	< 0.0001	6	476	5.8	1	0.016	2	482	1.92	1	0.1662
	Microscopy	277	186				0	463				0	463			

The overall frequency of non-*falciparum* malaria (*P. malariae* and *P. ovale*) detected by PCR was 122/5260 (2.3%), with *P. malariae* accounting for 76 of the 122 (62.3%) samples PCR-positive for non-*falciparum* malaria. The frequency of *P. malariae* was highest in the Ashanti region (15/519, 3.0%) and lowest in the Western region (6/484, 1.2%), with no *P. malariae* infection detected in the Greater Accra region. The frequency of *P. ovale* ranged from 2/655 (0.3%) in the Upper West region to 13/563 (2.3%) in the Brong Ahafo region (Fig. 1b).

##### Mixed species infections

The majority (109/122, 89.3%) of the non-*falciparum* malaria infections detected by PCR were detected as mixed infections. The overall frequency of *P. falciparum–P. malariae* (PfPm), *P. falciparum–P. ovale* (PfPo), *P. malariae–P. ovale* (PmPo) and *P. falciparum–P. malariae–P. ovale* (PfPmPo) mixed infections were 64/109 (58.7%), 42/109 (38.5%), 1/109 (0.9%) and 2/109 (1.8%) respectively.

The only case of infection that contained all three parasite species was observed in the Ashanti region, and the only case of *PmPo* mixed infection was observed in the Western region of Ghana. The highest frequency of PfPo infection was observed in the Brong Ahafo region (13/42, 31%) and the lowest in the Western region (1/42, 2.4%). The frequency of *PfPm* mixed infection was high in the Volta (13/64, 20.3%), Ashanti (12/64, 18.8%), Brong Ahafo (12/64, 18.8%), Central (10/64 (15.6%) and Northern (8/64, 12.5%) regions. The mean frequency of PfPm mixed infections and PmPo mixed infections across the 10 regions of Ghana were 6.4 ± 1.6 and 4.20 ± 1.2, respectively (Fig. 1c). There was a significant difference in *P. malariae* and *P. ovale* malaria diagnosis by PCR compared to microscopic diagnosis in the Ashanti (*χ*^2^ = 11.27, *df* = 1, *P* = 0.0008 and* χ*^2^ = 7.6, *df* = 1,* P * = 0.0058, respectively) and Brong Ahafo (*χ*^2^ = 15.2, *df* = 1, * P* < 0.0001 and* χ*^2^ = 13.15, *df* = 1,* P* = 0.0003, respectively) regions, where these parasites were identified (Table 2).

##### Submicroscopic infections (PCR-positive but microscopy-negative)

The PCR estimates of *P. falciparum* (*χ*^2^ = 258.6, *df* = 1,* P* < 0.0001), *P. malariae *(*χ*^2^ = 709.4, *df* = 1,* P* < 0.0001) and * P. ovale* (*χ*^2^ = 424.5, *df* = 1,* P* < 0.0001) were significantly higher than the microscopy estimates across all the regions. A total of 33.8% (1776/5260) of the symptomatic *P. falciparum* malaria infections were submicroscopic. The Ashanti region contributed the least to the overall number of individuals with submicroscopic infections (85/1776, 17.1%).

The majority (75/120, 63.3%) of submicroscopic non-*falciparum* infections were caused by *P. malariae* (Table 1). Submicroscopic infections accounted for 100% of the non-*falciparum* infections detected in all regions except for the Ashanti region where submicroscopic *P. malariae* and *P. ovale* accounted for 14/15 (93.3%) and 10/11 (90.9%), respectively, of the total non-*falciparum* infections identified in those regions (Table 1).

### Distribution of malaria parasite frequency by PCR in the 10 regions of Ghana by age and gender

**Fig. 2:**
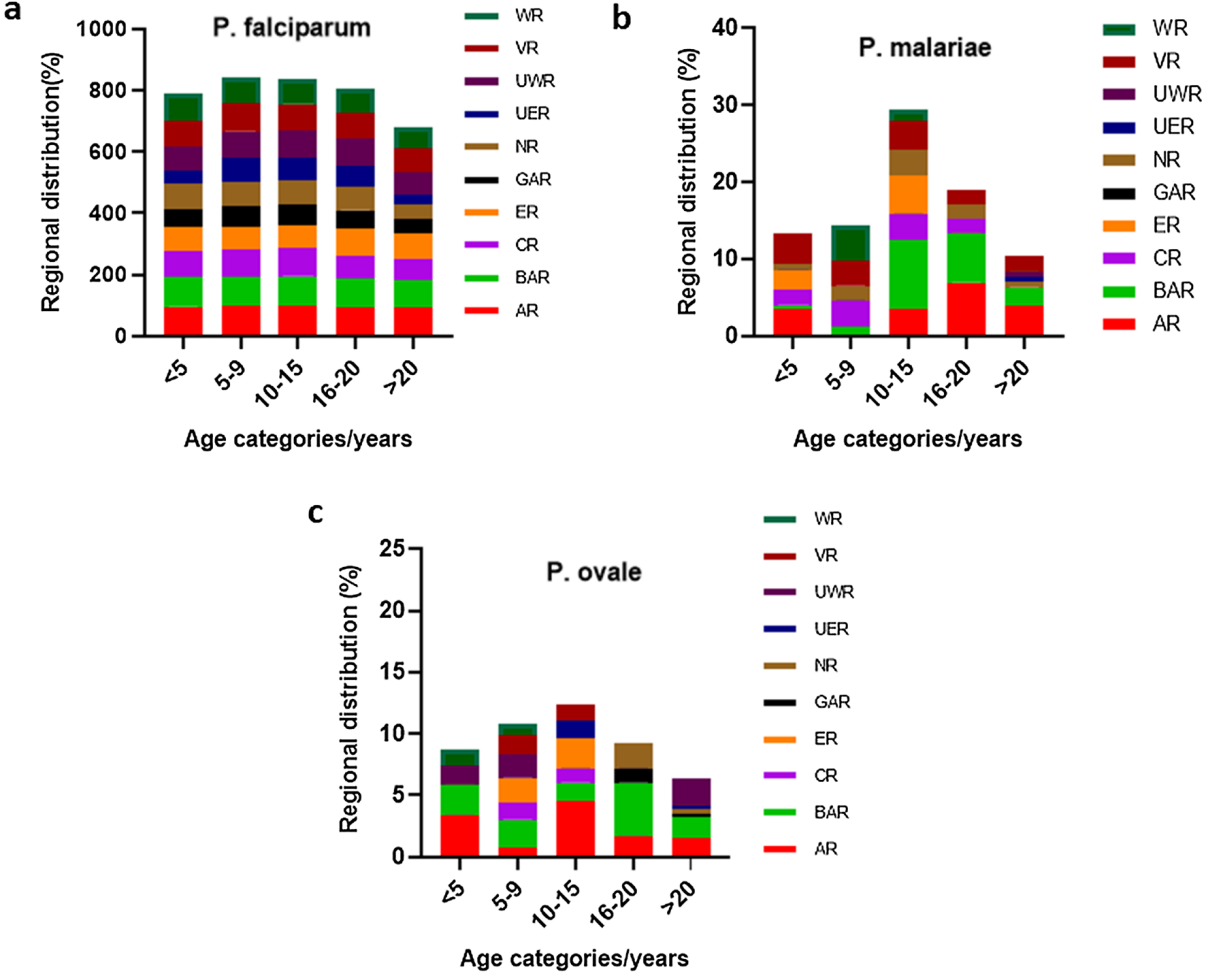
Regional distribution of *Plasmodium* species among age-stratified categories (< 5, 5–9, 10–15, 16–20 and > 20 years). **a** Regional distribution *of P. falciparum* in percentage across the age categories among the study subjects. **b** Regional distribution of* P. malariae* in percentage across the age categories among the study subjects. **c** Regional distribution of *P. ovale* in percentage across the age categories among the study subjects

The overall frequency of *P. falciparum* detected by PCR (Pf-PCR) among the age categories 0–4, 5–9, 10–15, 16–20 and > 20 years were 719/867 (82.9%), 987/1145 (86.2%), 591/700 (84.4%), 430/547 (78.6%) and 1135/1862 (61%), respectively. The Greater Accra and Eastern regions had the lowest Pf-PCR positivity rate among individuals aged 0–4 years (26/719, 3.6% and 23/719, 3.2%, respectively). The highest Pf-PCR positivity rate in individuals aged 0–4 years was identified in the Brong Ahafo region (150/719, 20.9%). There was a significantly lower frequency of malaria in individuals aged 10–15 years and 16–20 years compared to individuals aged 0–4 years (*χ*^2^ = 5.185, *df* = 1,* P* = 0.0228 and* χ*^2^ = 81.13, *df* = 1,* P* < 0.0001, respectively). However, individuals aged between 5 and 9 years had a higher frequency of malaria compared to individuals aged 0–4 years (*χ*^2^ = 20.48, *df* = 1,* P* < 0.0001 (Fig. 2a).

The overall frequency of *P. malariae* among the age group 10–15 years was 2.9% (20/700), with individuals from the Brong Ahafo region contributing the most in absolute numbers (6/20, 30%) within this age group. A total of 9.0% (6/67) of individuals in the age group 10–15 years from the Brong Ahafo region were infected with *P. malariae*. The frequency of *P. malariae* in individuals belonging to the age groups 16–20 years and > 20 years were highest in the Ashanti and Brong Ahafo regions (Fig. 2b).

A total of nine individuals aged 0–4 years were infected with *P. ovale*, with individuals from the Brong Ahafo region contributing the most (4/9, 44.4%) within this age group. In the Ashanti region, of the 88 individuals infected with *P. ovale*, three (3.4%) were aged 0–4 years.

A total of 13 individuals in the age group 10–15 years were infected with *P. ovale*, with individuals from the Brong Ahafo Region contributing the most (4/13, 30.8%) within this age group. Of the 86 individuals from the Ashanti Region aged between 10 and 15 years, four (4.7%) were infected with *P. ovale* (Fig. 2c).

Overall, there was a significant difference (*χ*^2^ = 33.702, *df* = 1,* P* < 0.0001) in the frequency of *P. falciparum* infections in male subjects (1667/2100, 79.4%) compared to female subjects (2195/3021, 72.7%). Male subjects had a relatively higher frequency of *P. falciparum* infections compared to their female counterparts across all the regions, with the exception of the Eastern region where PCR identified 80.7% (*n* = 134) of the overall female participants and 79.5% (*n* = 97) of the overall total male participants to be infected with *P. falciparum*.

**Fig. 3:**
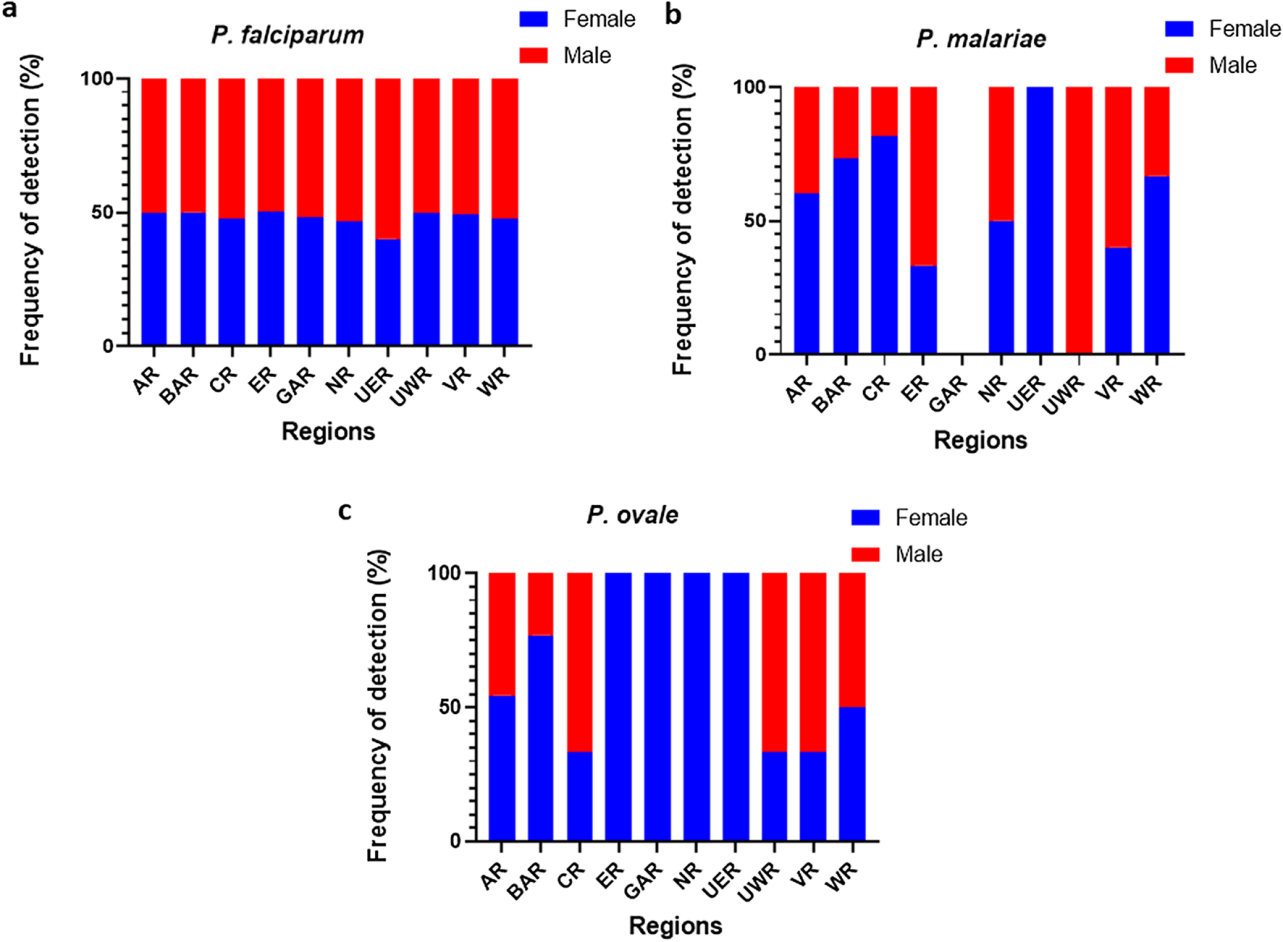
The distribution of *Plasmodium* species in male and female study participants in the 10 regions of Ghana. **a** Prevalence of *P. falciparum* infection among the male and female study subjects across the 10 regions of Ghana. **b** Pevalence of *P. malariae* infection among the male and female study subjects across the 10 regions of Ghana. **c** Prevalence of *P. ovale* infection among the male and female study subjects across the 10 regions of Ghana

*Plasmodium malariae* detected by PCR was identified in 46/3021 (1.5%) and 30/2100 (1.4%) of the female and male participants, respectively. The frequency of *P. malariae* was significantly higher (*χ*^2^ = 42.039, *df* = 1,* P* < 0.0001) among female participants than among male participants in all regions, except for the Eastern and Volta regions (Fig. 3b).

Overall, *P. ovale* was identified in 29/3021 (1.0%) and 17/2100 (0.8%) of female and male participants, respectively. Infections containing *P. ovale* were detected in only four regions. The frequency of *P. ovale* in the Ashanti region was similar between male (5/222, 2.3%) and female (6/286 (2.1%) participants. In the Brong Ahafo region, *P. ovale* was more prevalent in female (10/319, 3.1%) than males (3/241, 1.2%) participants. In the Eastern region, *P. ovale* was identified in 2/166 (1%) female subjects; in the Upper West region, 4/171 (2.3%) male subjects were identified with *P. ovale* (Fig. 3c).

## Discussion

Globally, recent years has seen a substantial reduction in clinical malaria incidence, morbidity and mortality [20, 21]. However, this achievement could be hindered by the presence of submicroscopic densities of *Plasmodium*, especially those that result in clinical disease. In Ghana, three main *Plasmodium* species cause malaria, namely *P. falciparum*, *P. malariae* and *P. ovale* [3, 22], and the main diagnostic tools used to identify malaria among symptomatic malaria patients are microscopy and HRP2 (histidine-rich protein 2)-based malaria RDT (rapid diagnostic test) kits. Our previous molecular studies on the composition of *Plasmodium* infections, including our study on school children in the Central region of Ghana and the community-based study in the Eastern region, focused predominantly on asymptomatic individuals [3, 22]. In the present study, we used PCR to determine the frequency of *Plasmodium* infections amongst suspected symptomatic malaria patients and identified patients with suspected malaria whose infection was missed by the routine point-of-care tool due to low parasite densities and also to HRP2 deletions since many RDTs are HRP2-based tests.

Although there has been a substantial reduction in malaria prevalence and mortality, there are reports of a high malaria burden in older children [23]. The present study identified a high frequency of *P. falciparum* and *P. ovale* in individuals aged between 5 and 15 years and an increased frequency of *P. malariae* in individuals aged between 10 and 20 years. The shift in malaria prevalence from younger children to older children could be due to the implementation of numerous malaria control interventions that have targeted vulnerable populations [24, 25]. In the present study, children aged < 5 years, an age group known to be the most risk of developing malaria [26, 27], did not have the highest frequency of malaria parasites. This finding suggests that the malaria control interventions which have been implemented for children aged < 5 years old are very effective and should be scaled up to include the entire population, starting with children aged 5–10 years who presented with the highest frequency of PCR-detectable malaria parasites in the present study. The results of this study also showed that the frequencies of *P. malariae* and *P. ovale* causing symptomatic disease are very similar to those reported previously from other countries in sub-Saharan Africa, where 0.2–4.8% *P. ovale* malaria cases and 0.3–2.8% of *P. malariae* malaria cases were associated with symptomatic malaria [14, 28–33].

While malaria affects both males and females [34, 35], gender was identified as a factor that influenced the vulnerability to malaria infection. The present study showed that males were significantly more likely to harbour a *P. falciparum* infection than female subjects, which could be the result of differences in exposure patterns between males and females. Males between the age of 5 and 15 years were more likely to have a greater risk of exposure to mosquito bites as they may stay longer outside without protection and be less likely to sleep under insecticide-treated bed nets [36]. From a public health perspective, the 7% difference in *P. falciparum* frequency between males and females may not influence the implementation of malaria control measures between males and females. However, it is worth noting as malaria parasite frequency in malaria-endemic communities is dynamic.

The frequency of *P. malariae* frequency identified in this study is similar to that reported previously from two regions of Ghana [37, 38]. Although the frequency of *P. malariae* was low, a large number of samples analysed provides the high statistical power to conclude the outcomes. The increased vulnerability of females to *P. malariae* is unknown; however, a similar observation was identified among females living in Orissa [37]. One possible explanation is that *P. malariae* is spread by indoor biting vectors and females have a higher exposure to indoor biting vectors [38, 39].

Point-of-care tools, including microscopy and malaria RDT kits, are required for malaria diagnosis to ensure that rapid and effective treatment can be provided. However, the sensitivity of these tools reduces significantly with decreasing parasite density of the infection [40]. The nested PCR assay used in this study identified a large proportion of malaria infections that were not detected by either or both microscopy and the malaria RDT and which remained either misdiagnosed or undiagnosed, similar to the previous reports from Cameroon [3, 40]. Submicroscopic malaria parasites among febrile patients have been enumerated; however, due to overlapping symptoms between malaria and other febrile diseases, highly sensitive POC diagnostic tests must be used for diagnosis [40]. Although submicroscopic malaria infections rarely cause clinical disease, in some cases severe and acute symptoms, such as mild anaemia, coughing, vomiting and jaundice, may develop as the disease progresses without appropriate treatment [41]. Early detection of submicroscopic malaria parasites is very important as it ensures appropriate treatment and the accurate determination of the malaria reservoir size [42, 43]. When submicroscopic malaria parasite infections are not detected and treated, they contribute to the maintenance of disease transmission as these infections frequently harbour gametocytes [44, 45].

The use of convenience sampling to obtain the samples for this study resulted in a bias, which caused the overall frequency of *Plasmodium* infections detected by microscopy (46.3%) in the subset of suspected malaria patients used in this study to be higher than that reported in the original larger study (16%) [17]. However, the focus of this study was not on determining the overall parasite prevalence per se but rather on the regional distribution of the various *Plasmodium* species and on highlighting the higher sensitivity of PCR at detecting malaria parasite infections, especially the non-*falciparum* species, amongst patients with suspected malaria attending healthcare facilities.

## Conclusion

*Plasmodium malariae* and *P. ovale* as mono-infections resulted in symptomatic malaria cases. *Plasmodium falciparum* and *P. ovale* were more commonly detected in children aged between 5 and 15 years, whilst *P. malariae* was more prevalent in individuals aged between 10 and 20 years. Male subjects had a higher frequency of *P. falciparum* infection than female subjects, whilst *P. malariae* was more prevalent in female subjects. More sensitive point-of-care tools are needed to detect the presence of low-density (submicroscopic) *Plasmodium* infections, which can result in symptomatic infections (Additional file 1).

## Supplementary Information

**Additional file 1: Figure S1.** Representative gel images of products from the different *Plasmodium* speciation reactions.

## Data Availability

All data generated or analysed during this study are included in this published article.

## References

[1] World Health Organization (WHO). World malaria report 2020: 20 years of global progress and challenges. 2020. https://apps.who.int/iris/handle/10665/337660. Accessed 2 Jan 2021.

[2] Crawley J, Chu C, Mtove G, Nosten F (2010). Malaria in children. Lancet.

[3] Amoah LE, Donu D, Abuaku B, Ahorlu C, Arhinful D, Afari E (2019). Probing the composition of *Plasmodium* species contained in malaria infections in the Eastern region of Ghana. BMC Public Health.

[4] Awine T, Malm K, Bart-Plange C, Silal SP (2017). Towards malaria control and elimination in Ghana: challenges and decision-making tools to guide planning. Glob Health Action.

[5] Amir A, Cheong FW, De Silva JR, Lau YL (2018). Diagnostic tools in childhood malaria. Parasit Vectors.

[6] Iwuafor AA, Ita OI, Ogban GI, Udoh UA, Amajor CA (2018). Evaluation of diagnostic accuracy of rapid diagnostic test for malaria diagnosis among febrile children in Calabar, Nigeria. Niger Med J.

[7] Berzosa P, de Lucio A, Romay-Barja M, Herrador Z, González V, García L (2018). Comparison of three diagnostic methods (microscopy, RDT, and PCR) for the detection of malaria parasites in representative samples from Equatorial Guinea. Malar J.

[8] Loick Pradel KF, Pande V, Singh V (2021). Field performances of rapid diagnostic tests detecting human Plasmodium species: a systematic review and meta-analysis in India, 1990–2020. Diagnostics.

[9] Jiram AI, Ooi CH, Rubio JM, Hisam S, Karnan G, Sukor NM (2019). Evidence of asymptomatic submicroscopic malaria in low transmission areas in Belaga district, Kapit division, Sarawak, Malaysia. Malar J.

[10] Imbert P, Rapp C, Buffet PA (2009). Pathological rupture of the spleen in malaria: analysis of 55 cases (1958–2008). Travel Med Infect Dis.

[11] Haanshuus CG, Mørch K, Blomberg B, Strøm GE, Langeland N, Hanevik K (2019). Assessment of malaria real-time PCR methods and application with focus on low-level parasitaemia. PLoS ONE.

[12] Heinemann M, Phillips RO, Vinnemeier CD, Rolling CC, Tannich E, Rolling T (2020). High prevalence of asymptomatic malaria infections in adults, Ashanti Region, Ghana, 2018. Malar J.

[13] Cao Y, Wang W, Liu Y, Cotter C, Zhou H, Zhu G (2016). The increasing importance of *Plasmodium ovale* and *Plasmodium malariae* in a malaria elimination setting: an observational study of imported cases in Jiangsu Province, China, 2011–2014. Malar J.

[14] Kotepui M, Kotepui KU, Milanez GD, Masangkay FR (2020). Severity and mortality of severe *Plasmodium ovale* infection: a systematic review and meta-analysis. PLoS ONE.

[15] Li P, Zhao Z, Wang Y, Xing H, Parker DM, Yang Z (2014). Nested PCR detection of malaria directly using blood filter paper samples from epidemiological surveys. Malar J.

[16] Amoah LE, Abuaku B, Bukari AH, Dickson D, Amoako EO, Asumah G (2020). Contribution of P falciparum parasites with Pfhrp 2 gene deletions to false negative PfHRP 2 based malaria RDT results in Ghana: a nationwide study of symptomatic malaria patients. PLoS ONE.

[17] Abuaku B, Amoah LE, Peprah NY, Asamoah A, Amoako EO, Donu D (2021). Malaria parasitaemia and mRDT diagnostic performances among symptomatic individuals in selected health care facilities across Ghana. BMC Public Health.

[18] Amoah LE, Opong A, Ayanful-Torgby R, Abankwa J, Acquah FK (2016). Prevalence of G6PD deficiency and *Plasmodium falciparum* parasites in asymptomatic school children living in southern Ghana. Malar J.

[19] Obboh EK, Okonu RE, Amoah LE (2020). Large variations in malaria parasite carriage by afebrile school children living in nearby communities in the central region of Ghana. J Trop Med.

[20] Weiss DJ, Lucas TC, Nguyen M, Nandi AK, Bisanzio D, Battle KE (2019). Mapping the global prevalence, incidence, and mortality of *Plasmodium falciparum*, 2000–17: a spatial and temporal modelling study. Lancet.

[21] Battle KE, Lucas TC, Nguyen M, Howes RE, Nandi AK, Twohig KA (2019). Mapping the global endemicity and clinical burden of *Plasmodium vivax*, 2000–17: a spatial and temporal modelling study. Lancet.

[22] Bredu D, Donu D, Amoah LE (2021). Dynamics of the composition of Plasmodium species contained within asymptomatic malaria infections in the central region of Ghana. J Trop Med.

[23] Griffin JT, Ferguson NM, Ghani AC (2014). Estimates of the changing age-burden of *Plasmodium falciparum* malaria disease in sub-Saharan Africa. Nat Commun.

[24] Azabre BA, Teye JK, Yaro JA (2013). Malaria control strategies in the Kassena-Nankana east and west districts of Ghana. Ghana J Geography.

[25] Monroe A, Asamoah O, Lam Y, Koenker H, Psychas P, Lynch M (2015). Outdoor-sleeping and other night-time activities in northern Ghana: implications for residual transmission and malaria prevention. Malar J.

[26] Winskill P, Walker PG, Cibulskis RE, Ghani AC (2019). Prioritizing the scale-up of interventions for malaria control and elimination. Malar J.

[27] World Health Organization (WHO). Achieving and maintaining universal coverage with long-lasting insecticidal nets for malaria control. 2017. https://apps.who.int/iris/handle/10665/259478. Accessed 7 Mar 2021.

[28] Yman V, Wandell G, Mutemi DD, Miglar A, Asghar M, Hammar U (2019). Persistent transmission of *Plasmodium malariae* and *Plasmodium ovale* species in an area of declining *Plasmodium falciparum* transmission in eastern Tanzania. PLoS Negl Trop Dis.

[29] Akala HM, Watson OJ, Mitei KK, Juma DW, Verity R, Ingasia LA (2021). *Plasmodium* interspecies interactions during a period of increasing prevalence of *Plasmodium ovale* in symptomatic individuals seeking treatment: an observational study. Lancet Microbe.

[30] Djallé D, Gody JC, Moyen JM, Tekpa G, Ipero J, Madji N (2014). Performance of Paracheck™-Pf, SD Bioline malaria Ag-Pf and SD Bioline malaria Ag-Pf/pan for diagnosis of *falciparum* malaria in the Central African Republic. BMC Infect Dis.

[31] Culleton RL, Mita T, Ndounga M, Unger H, Cravo PV, Paganotti GM (2008). Failure to detect *Plasmodium vivax* in West and Central Africa by PCR species typing. Malar J.

[32] Langford S, Douglas NM, Lampah DA, Simpson JA, Kenangalem E, Sugiarto P (2015). *Plasmodium malariae* infection associated with a high burden of anemia: a hospital-based surveillance study. PLoS Negl Trop Dis.

[33] Ricci F (2012). Social implications of malaria and their relationships with poverty. Mediterr J Hematol..

[34] Pell C, Straus L, Andrew EV, Meñaca A, Pool R (2011). Social and cultural factors affecting uptake of interventions for malaria in pregnancy in Africa: a systematic review of the qualitative research. PLoS ONE.

[35] Njatosoa AF, Mattern C, Pourette D, Kesteman T, Rakotomanana E, Rahaivondrafahitra B (2021). Family, social and cultural determinants of long-lasting insecticidal net (LLIN) use in Madagascar: secondary analysis of three qualitative studies focused on children aged 5–15 years. Malar J.

[36] Dhangadamajhi G, Ranjit MR (2009). High prevalence and gender bias in distribution of *Plasmodium malariae* infection in central east-coast India. Trop Biomed.

[37] Steenkeste N, Rogers WO, Okell L, Jeanne I, Incardona S, Duval L (2010). Sub-microscopic malaria cases and mixed malaria infection in a remote area of high malaria endemicity in Rattanakiri province, Cambodia: implication for malaria elimination. Malar J.

[38] Ototo EN, Mbugi JP, Wanjala CL, Zhou G, Githeko AK, Yan G (2015). Surveillance of malaria vector population density and biting behaviour in western Kenya. Malar J.

[39] Doumbe-Belisse P, Ngadjeu CS, Sonhafouo-Chiana N, Talipouo A, Djamouko-Djonkam L, Kopya E (2018). High malaria transmission sustained by Anopheles gambiae sl occurring both indoors and outdoors in the city of Yaoundé. Cameroon Wellcome Open Res.

[40] Mfuh KO, Achonduh-Atijegbe OA, Bekindaka ON, Esemu LF, Mbakop CD, Gandhi K (2019). A comparison of thick-film microscopy, rapid diagnostic test, and polymerase chain reaction for accurate diagnosis of *Plasmodium falciparum* malaria. Malar J.

[41] Whittaker C, Slater H, Nash R, Bousema T, Drakeley C, Ghani AC (2021). Global patterns of submicroscopic *Plasmodium falciparum* malaria infection: insights from a systematic review and meta-analysis of population surveys. Lancet Microbe.

[42] Gerardin J, Ouédraogo AL, McCarthy KA, Eckhoff PA, Wenger EA (2015). Characterization of the infectious reservoir of malaria with an agent-based model calibrated to age-stratified parasite densities and infectiousness. Malar J.

[43] Gonçalves BP, Kapulu MC, Sawa P, Guelbéogo WM, Tiono AB, Grignard L (2017). Examining the human infectious reservoir for *Plasmodium falciparum* malaria in areas of differing transmission intensity. Nat Commun.

[44] Singh US, Praharaj M, Sharma C, Das A (2016). Paradigm shift in transmission of vector-borne diseases. Emerg Infect Dis.

[45] Varo R, Balanza N, Mayor A, Bassat Q (2021). Diagnosis of clinical malaria in endemic settings. Expert Rev Anti Infect.

