# Supplementary material for: Nationwide molecular surveillance of three Plasmodium species harboured by symptomatic malaria patients living in Ghana
Source: Parasit Vectors. 2022 Jan 28;15:40. doi: 10.1186/s13071-022-05153-6 (PMC8796507; doi:10.1186/s13071-022-05153-6)
Supplement: Supplementary file 1 — Additional file 1: Figure S1. Representative gel images of products from the different Plasmodium speciation reactions. [file 13071_2022_5153_MOESM1_ESM.pdf]

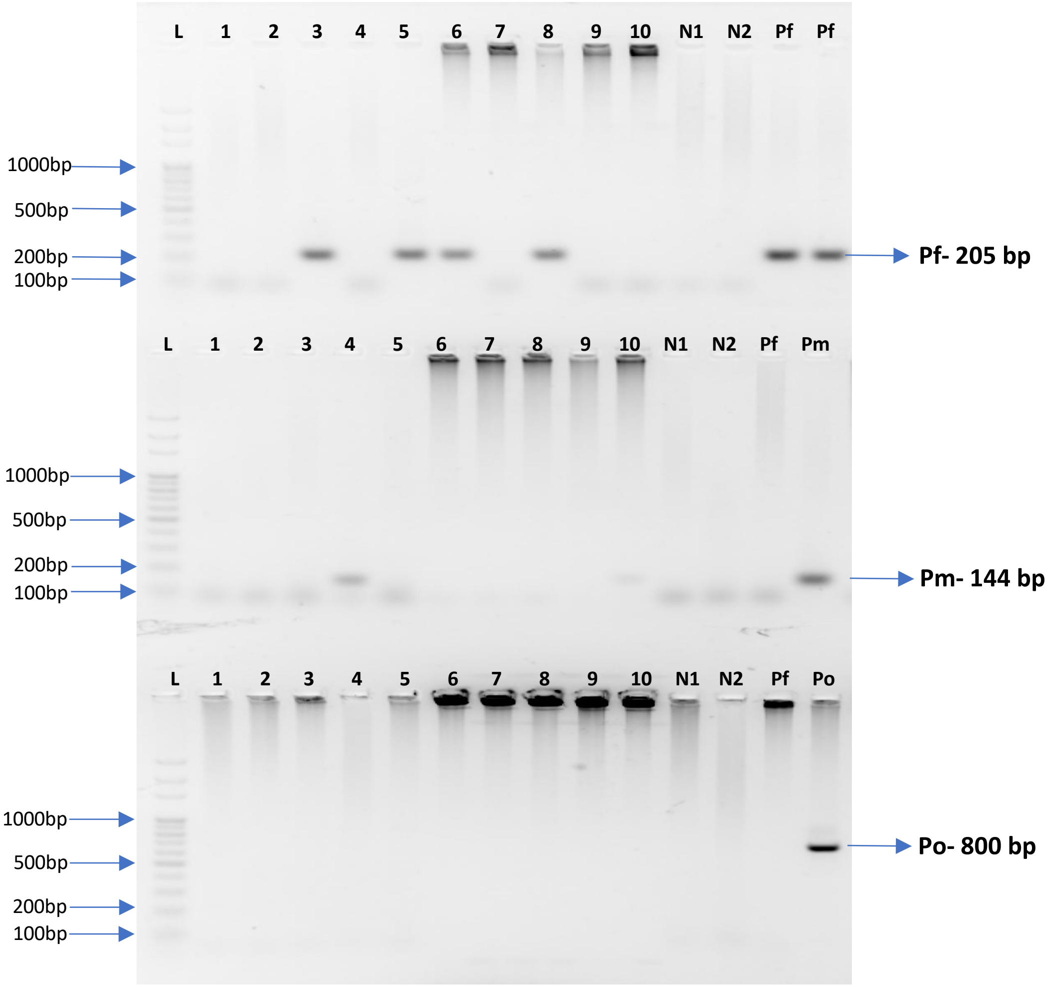

2% Agarose Gel

LEGEND

- Lane 1- 100 bp Ladder
- Lane 2-11 : Field samples
- Lane 12-13: Negative Controls
- Lane 14-15: Positive Controls
